# Physiological and Molecular Response Mechanisms of *Betaphycus gelatinus* to Low- and High-Temperature Stress

**DOI:** 10.3390/ijms27020593

**Published:** 2026-01-07

**Authors:** Yongqiu Deng, Siqi Xu, Kangtai Liao, Linwen He

**Affiliations:** School of Marine Biology and Fisheries, Hainan University, Haikou 570228, China; dengyongqiu2025@hainanu.edu.cn (Y.D.); xusiqi2025@hainanu.edu.cn (S.X.); liaokangtai0206@hainanu.edu.cn (K.L.)

**Keywords:** *Betaphycus gelatinus*, temperature stress, photosynthesis, omics

## Abstract

*Betaphycus gelatinus*, a member of the Eucheumatoideae, serves as the primary source for carrageenan extraction and has significant economic value. The growth and reproduction of *B. gelatinus* are significantly impacted by seasonal fluctuations in seawater temperature. To explore its adaptive mechanisms under temperature stress, we cultured the algae at 15 °C (Low temperature, LT), 27 °C (Medium temperature, MT), and 36 °C (High temperature, HT) for 2 h and conducted subsequent physiological, transcriptomics, and metabolomics analyses. The photosynthetic performance of *B. gelatinus* significantly declined under both LT and HT stress conditions. Carotenoid content increased significantly under LT conditions, while chlorophyll a showed no significant change. Phycocyanin and phycoerythrin decreased significantly under LT conditions, but there was no significant difference under HT conditions. Under LT stress, glutathione (GSH) levels, ascorbate peroxidase (APX) activity, and catalase (CAT) activity all increased significantly. Under HT stress, APX and CAT activities increased significantly, while superoxide dismutase (SOD) activity and malondialdehyde (MDA) levels remained unchanged. Transcriptomics and metabolomics analyses suggested that photosynthesis, carbohydrate metabolism, amino acid biosynthesis, porphyrin metabolism, and vitamin B6 metabolism are involved in the acute temperature stress response of *B. gelatinus.* Under both HT and LT, most genes in the targeted metabolic pathways were significantly downregulated (*p* < 0.05), while only a few were upregulated. Specifically, in carbohydrate metabolism, only nine genes were upregulated, while all others were downregulated. Moreover, all the genes involved in photosynthesis, photosynthetic carbon fixation, arginine biosynthesis, and porphyrin metabolism were downregulated. In contrast, only four genes involved in GSH metabolism, alanine, aspartate, and glutamate metabolism, and glycine, serine, and threonine metabolism were upregulated. These results suggest that temperature stress markedly suppresses the transcription of key genes in these pathways and that the few upregulated genes in these pathways may contribute to compensatory mechanisms or regulatory network reprogramming during stress responses. These findings help clarify how *B. gelatinus* adapts to different temperature stresses and provide a basis for developing improved germplasm to support stable production under climate variability.

## 1. Introduction

Algae have emerged as one of the most promising biological resources due to their remarkable adaptability and resource sustainability. In recent decades, the global algal market has experienced significant expansion. According to FAO data, global seaweed output increased nearly threefold from 118,000 tons in 2000 to 358,200 tons in 2019, with 97% of this production originating from aquaculture. The economic value of algal products is equally substantial; the market was valued at approximately $4 billion in 2018. This growth is driven by the increasing demand for high-value metabolites such as carotenoids and bioactive polysaccharides in the food, pharmaceutical, and cosmetic industries [1,2]. *Betaphycus gelatinus* (formerly *Eucheuma gelatinum*) is a large, economically significant red alga, particularly valued for its carrageenan content [3]. It also contains abundant nutrients, including proteins, lipids, carbohydrates, α-tocopherol, minerals, and various vitamins. It has served as the primary raw material for carrageenan production for over three decades [4,5]. However, it exhibits pronounced seasonal variations in growth rate and biochemical composition, which are influenced by environmental variables such as temperature, salinity, light levels, and nutrient supply [6,7].

Seasonal changes cause substantial fluctuations in seawater temperature. For instance, increased solar radiation in summer rapidly elevates the surface temperature, whereas reduced sunlight in winter decreases it [8]. Such fluctuations strongly influence the growth and metabolism of marine algae [9]. Macroalgal photosynthetic rates are largely determined by environmental temperature [10]. When exposed to extreme temperatures, algal thalli undergo oxidative stress, thereby increasing reactive oxygen species (ROS) levels and leading to injury of cell membranes, proteins, and lipids. Throughout evolution, algae have acquired a dual antioxidant strategy, relying on both enzymatic defenses—SOD, CAT, APX, and glutathione reductase—and non-enzymatic compounds such as carotenoids, tocopherols, and glutathione to reduce oxidative damage [11]. They exhibit dynamic gene expression changes alongside physiological responses, involving multiple metabolic pathways like photosynthesis, energy metabolism, carbohydrate metabolism [12], lipid metabolism [13], and amino acid metabolism [14,15]. Understanding algal responses to extreme temperatures can provide valuable insights for aquaculture and environmental protection.

Most existing research on *B. gelatinus* has concentrated on the physiological and biochemical characteristics. The optimal growth temperature range for *B*. *gelatinus* is 24–28 °C, with an optimum growth temperature of 27 °C [7,16]. Its growth rate is significantly reduced at temperatures lower than 16 °C and higher than 30 °C [16]. Omics approaches are crucial for elucidating the molecular mechanisms underlying red algal responses to environmental stressors, as observed for *Kappaphycus alvarezii* and *Eucheuma denticulatum* [17,18]. These studies provide a theoretical basis for exploring temperature stress responses in *B. gelatinus*. Existing omics studies on *B. gelatinus* are limited to salinity stress analysis [19], while the molecular mechanisms governing its acute temperature stress response remain unclear.

In this study, *B. gelatinus* was treated at 15 °C (low temperature; LT), 27 °C (medium temperature; MT), and 36 °C (high temperature; HT) for 2 h, and its photosynthetic performance, pigment content, GSH and malondialdehyde (MDA) levels, and SOD, CAT, and APX activities were measured. Transcriptomic and metabolomic analyses were also performed on the three treatments. These results will advance our comprehension of the molecular and physiological processes involved in *B. gelatinus* adaptation to temperature fluctuations.

## 2. Results

### 2.1. Physiological Change

**Figure 1:**
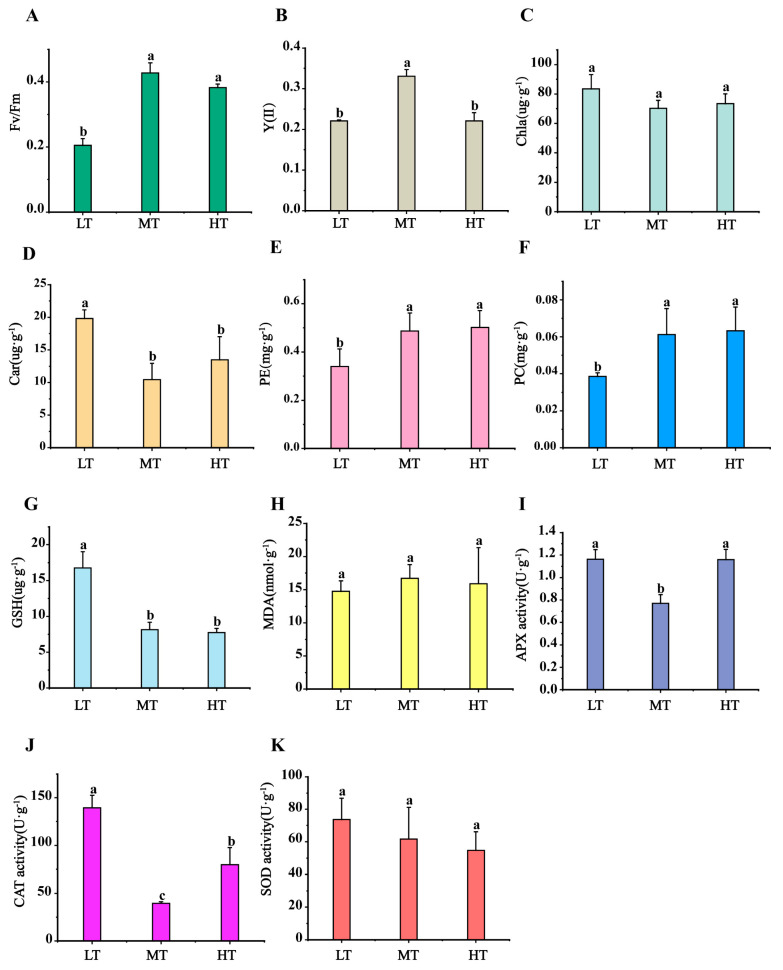
Changes in (**A**) maximum photosynthetic quantum yield of PSII (Fv/Fm), (**B**) effective photochemical quantum yield of PSII (Y(II)), (**C**) Chlorophyll a (Chl a) content, (**D**) Carotenoid (Car) content, (**E**) Phycoerythrin (PE) content, (**F**) Phycocyanin (PC) content, (**G**) glutathione (GSH) content, (**H**) Malondialdehyde (MDA) content, (**I**) ascorbate peroxidase (APX) activity, (**J**) Catalase Activity, (**K**) superoxide dismutase (SOD) in *B. gelatinus* under different temperature conditions. Means and SD are shown, *n* = 3 in (**A**–**K**), the data of each treatment were analyzed using one-way analysis of variance (ANOVA), and the significance of differences between the means were detected using Duncan’s multiple range (*p* < 0.05), different lowercase letters indicate significant differences (*p* < 0.05) between different temperature treatments.

Physiological responses of *B. gelatinus* to temperature stress were analysed with a focus on photosynthetic activity, pigment concentrations, MDA levels, antioxidant enzyme activities, and antioxidant compound contents (Figure 1A–K). Compared to MT (control), the Fv/Fm ratio decreased significantly under LT stress (0.20 ± 0.02; *p* < 0.05; Figure 1A), but showed no significant change under HT stress. A significant decrease in Y(II) values was observed under both LT and HT conditions compared to the control (Figure 1B). Chl a content did not vary significantly from the control under both stress conditions, whereas Car content increased markedly under LT (19.82 ± 1.32 mg·g^−1^). The content of PE (phycoerythrin) and PC (Phycocyanin) decreased significantly under LT conditions, while no significant changes were observed under HT conditions. (Figure 1C–F). GSH content increased considerably to 16.75 ± 2.26 μg·g^−1^ under LT compared to MT (8.14 ± 1.04 μg·g^−1^), but remained unchanged under HT (Figure 1G). Malondialdehyde (MDA) levels did not change significantly under either temperature stress (Figure 1H). Furthermore, APX and CAT activities increased significantly under temperature stress, with CAT activity being significantly elevated under both LT and HT conditions compared to MT (LT: 139.44 ± 12.98 U·g^−1^, MT: 39.55 ±1.71 U·g^−1^, HT: 79.78 ± 17.89 U·g^−1^) (Figure 1J). SOD activity did not experience any substantial variations under LT/HT stress.

### 2.2. Transcriptome and Metabolome Response Under Temperature Stress

Transcriptome sequencing was conducted for three biological replicates of each LT, MT, and HT group. A total of 64.72 GB of high-quality clean data was yielded following quality control, with Q30 values exceeding 95.48% in all samples (Table S1). De novo assembly generated 35,521 transcript sequences and 27,119 unigenes, with N50 values of 4564 and 3246 bp, respectively (Table S2). Successful functional annotation of 11,372 assembled unigenes was performed using the Pfam, GO, KEGG, NR, Swiss-Prot, and eggNOG databases in the DIAMOND software(V2.1.9), with 9237 (34.06%), 8086 (29.82%), 7482 (27.59%), 8958 (33.03%), 8373 (30.88%), and 9737 (35.90%) unigenes annotated in the Pfam, GO, KEGG, NR, Swiss-Prot, and eggNOG databases, respectively (Table S3). PCA and Pearson correlation analysis revealed high intra-group correlations and significant differences between the LT/HT treatment groups and the control (Figure S1).

Compared to the MT group, 1292 DEGs (501 upregulated and 791 downregulated) were identified in the LT group and 1101 DEGs (422 upregulated and 679 downregulated) in the HT group (FC ≥ 2; FDR < 0.05) (Figure S2), with 351 between the two (Figure S3). qRT-PCR and mRNA-Seq analyses showed consistent expression trends for nine randomly selected DEGs (Figure S4), verifying the sequencing results. The most significantly enriched BP, CC, and MF terms in both LT vs. MT and HT vs. MT groups were related to photosynthetic light harvesting, photosystem I, and Chl binding (Figure S5). KEGG enrichment analysis indicated that, in contrast to the MT group, the DEGs in the LT group showed significant enrichment in the pentose phosphate pathway (PPP; map00030); porphyrin metabolism (map00860); photosynthetic carbon fixation (map00710); fructose and mannose metabolism (map00051); photosynthesis-antenna proteins (map00196); alanine, aspartate, and glutamate (Ala-Asp-Glu) metabolism (map00250); fatty acid elongation (map00062); and glycine, serine, and threonine (Gly-Ser-Thr) metabolism (map00260) and (ii) HT group were significantly enriched in glycolysis/gluconeogenesis (map00010), photosynthesis (map00195), photosynthetic carbon fixation (map00710), PPP (map00030), porphyrin metabolism (map00860), fructose and mannose metabolism (map00051), photosynthesis-antenna proteins (map00196), arginine (Arg) biosynthesis (map00220), and starch and sucrose metabolism (map00500) (Figure S2, Table S4).

Compared to the MT group, 72 and 64 differential metabolites were identified in the LT (33 upregulated and 39 downregulated) and HT (38 upregulated and 26 downregulated) groups, respectively (*n* = 6 per group). KEGG enrichment analysis revealed that compared to the MT, the DAMs in the LT group exhibited substantial enrichment in pathways linked to cofactor biosynthesis (map01240), pantothenate and CoA biosynthesis (map00770), and ABC transporters (map02010); and HT group were significantly enriched in purine metabolism (map00230), Ala-Asp-Glu metabolism (map00250), nucleotide metabolism (map01232), and ABC transporters (map02010), (Figure S6; Table S5). Among these, 22 differential metabolites were shared between the LT and HT groups (Figure S7).

### 2.3. Effects of Temperature Stress on Photosynthesis

**Figure 2:**
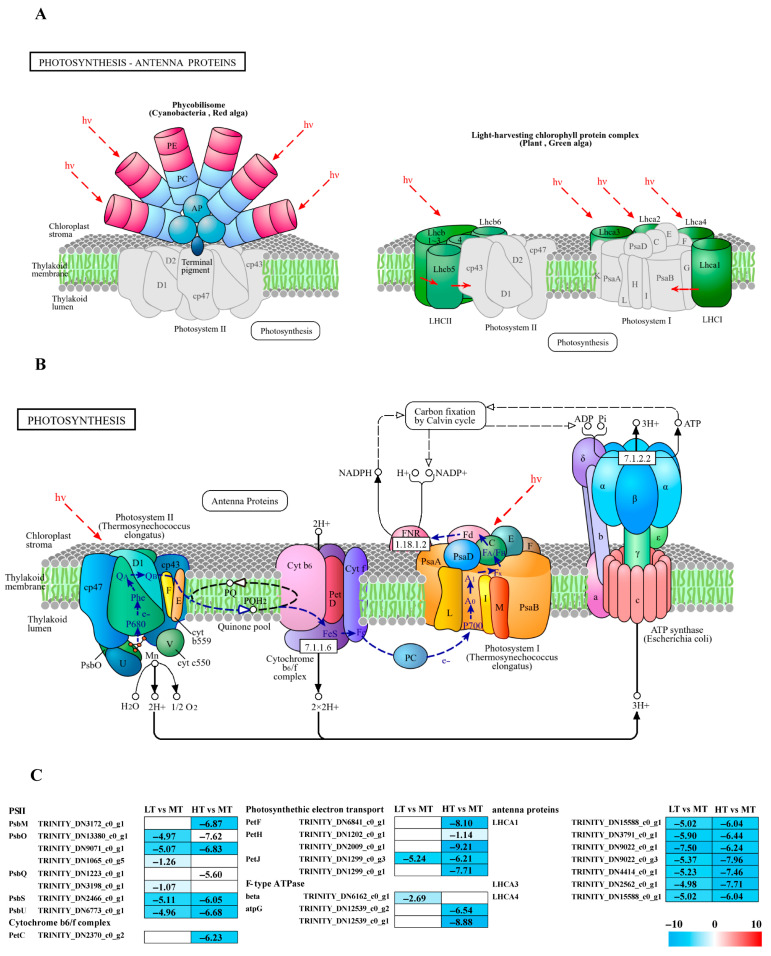
The transcriptomic and metabolomic changes in the photosynthesis and photosynthetic antenna proteins of *B. gelatinus* under different temperature conditions: (**A**) photosynthetic antenna protein pathway (ko00196), the solid arrows represent molecular interactions or relations, while the dashed arrows indicate indirect links or unknown reactions. (**B**) photosynthesis pathway (ko00195), (**C**) DEGs involved in photosynthesis and photosynthetic antenna proteins. The number in each cell is the log_2_ fold change (log_2_FC). Red and blue gradients indicate the upregulation and downregulation of unigenes, respectively, while the white color represents log_2_FC < 1 or FDR > 0.05. The diagrams of photosynthesis and photosynthetic antenna proteins were obtained from the KEGG website Gene abbreviations are defined in Supplementary Table S6.

14 DEGs associated with photosynthesis were identified, which contribute to the formation of photosystem II components such as *PsbM*, *PsbO*, *PsbQ*, *PsbS*, and *PsbU*, as well as the cytochrome b6/f complex (*PetC*), and F-type ATPase (β and γ subunits), along with electron transport (*PetF*, *PetH*, and *PetJ*) (Figure 2B). All these DEGs were significantly downregulated in both LT and HT groups compared to the control (Figure 2C). Additionally, 7 DEGs linked to photosynthetic antenna proteins, specifically light-harvesting complex I Chl a/b-binding proteins (*LHCA1*, *LHCA3*, and *LHCA4*), were identified and significantly downregulated in both extreme conditions (Figure 2A).

### 2.4. Effects of Temperature Stress on Carbohydrate and Energy Metabolism

**Figure 3:**
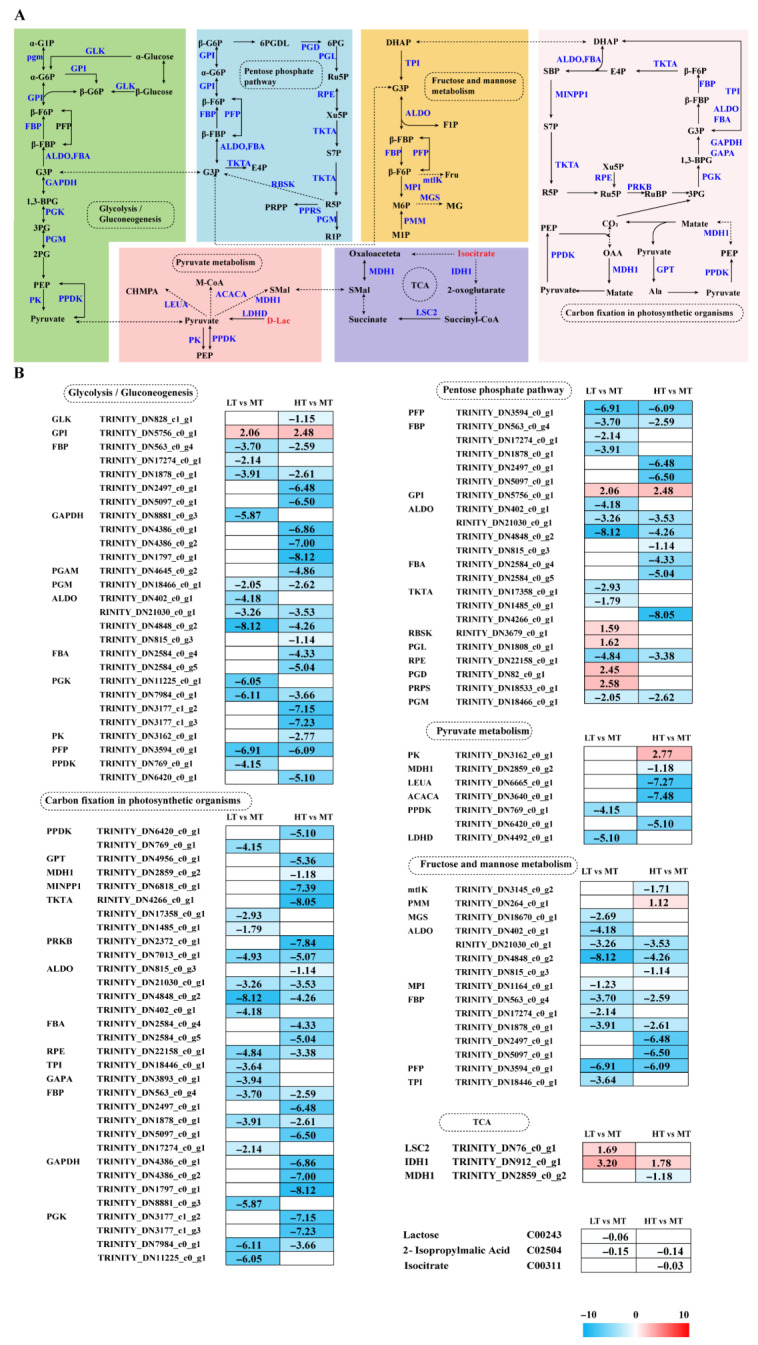
The transcriptomic and metabolomic changes in the Glycolysis/Gluconeogenesis, Pentose phosphate pathway, Fructose and mannose metabolism, TCA cycle, and Pyruvate metabolism of *B. gelatinus* under different temperature conditions. (**A**) Network diagram of carbohydrate metabolism pathways, different colors correspond to the distinct biological pathways, the solid arrows represent molecular interactions or relations, while the dashed arrows indicate indirect links or unknown reactions. (**B**) Differentially expressed genes (DEGs) and differential metabolites (DAMs) related to Glycolysis/Gluconeogenesis, Pentose phosphate pathway, Fructose and mannose metabolism, TCA cycle, Pyruvate metabolism. Red represents annotated differential metabolites, and blue represents annotated DEGs. The number in each cell is the log2 fold change (log_2_FC). Red and blue gradients indicate the upregulation and downregulation of unigenes and metabolites, respectively, while the white color represents a DEG (log_2_FC < 1 or FDR > 0.05) or a DM (FC < 1, *p*-value > 0.05 and VIP < 1). Gene abbreviations are defined in Supplementary Table S6.

Transcriptome analysis revealed DEGs notably enriched in carbohydrate and energy metabolism pathways, strongly influencing the response of *B. gelatinus* to temperature changes (Figure 3A). Under LT stress, five genes enriched in the PPP, namely *GPI*, *RBSK*, *PGL*, *PGD*, and *PRPS*, were significantly upregulated, whereas genes encoding *PFP*, *ALDO*, *TKT*, *RPE*, and *PGM* were significantly downregulated. Under HT stress, only the gene responsible for encoding *GPI* exhibited a notable increase in regulation, whereas other genes (*PFP*, *FBP*, *ALDO*, *FBA*, *TKT*, etc.) were significantly downregulated. The enzymes encoded by the genes that were downregulated primarily played a role in the non-oxidative phase or glycolysis, suggesting inhibition of these pathways under HT conditions. Key genes in glycolysis and gluconeogenesis (e.g., *GPI*, *FBP*, *GAPDH*, and *ALDO*) exhibited significant downregulation in response to both LT and HT. *GPI* was significantly upregulated in both groups, indicating that selective enhancement of specific steps may slow overall energy metabolism. Additionally, *GPI* upregulation may contribute to ROS scavenging due to its non-enzymatic functions, protecting cells from oxidative damage. In LT conditions, six DEGs (*MTLK*, *MGS*, *MPI*, *TPI*, *FBP*, and *ALDO*) were significantly downregulated in fructose and mannose metabolism. Similarly, in HT conditions, except for the significantly upregulated *PMM* gene, all other genes (*mtlK*, *ALDO*, *FBP*, and *PFP*) were significantly downregulated. *PMM* upregulation promotes adjustment of cellular metabolism toward glycolysis (EMP) and the PPP.

The core reactions of fructose and mannose metabolism directly produce dihydroxyacetone phosphate (DHAP), which subsequently enters cellular energy metabolism. During photosynthetic carbon fixation, all enriched DEGs (e.g., *GAPDH*, *PGK*, *PPDK*, *TKT*, *PRKB*, *RPE*, and *GAPA*) were significantly downregulated under LT stress. Similarly, under HT stress, all corresponding genes (e.g., *GAPDH*, *PGK*, *PPDK*, *GPT*, *MDH1*, *PRKB*, and *ALDO*) were significantly downregulated, indicating severely impaired algal carbon assimilation. Additionally, under LT stress, two TCA cycle-related genes, i.e., *LSC2* and *IDH*, were significantly upregulated, whereas two pyruvate metabolism genes, i.e., *PPDK* and *LDHD*, were significantly downregulated. Under HT stress, among TCA cycle genes, *IDH1* was upregulated and *MDH1* was downregulated. Most enriched pyruvate metabolism genes, such as *PPDK*, *MDH1*, *LEUA*, and *ACACA*, were downregulated, while *PK* was upregulated. Metabolomics analysis further revealed that under LT stress, lactate (Lac) was downregulated, whereas under HT stress, isocitrate was downregulated (Figure 3B).

### 2.5. Effects of Temperature Stress on Amino Acid Biosynthesis

**Figure 4:**
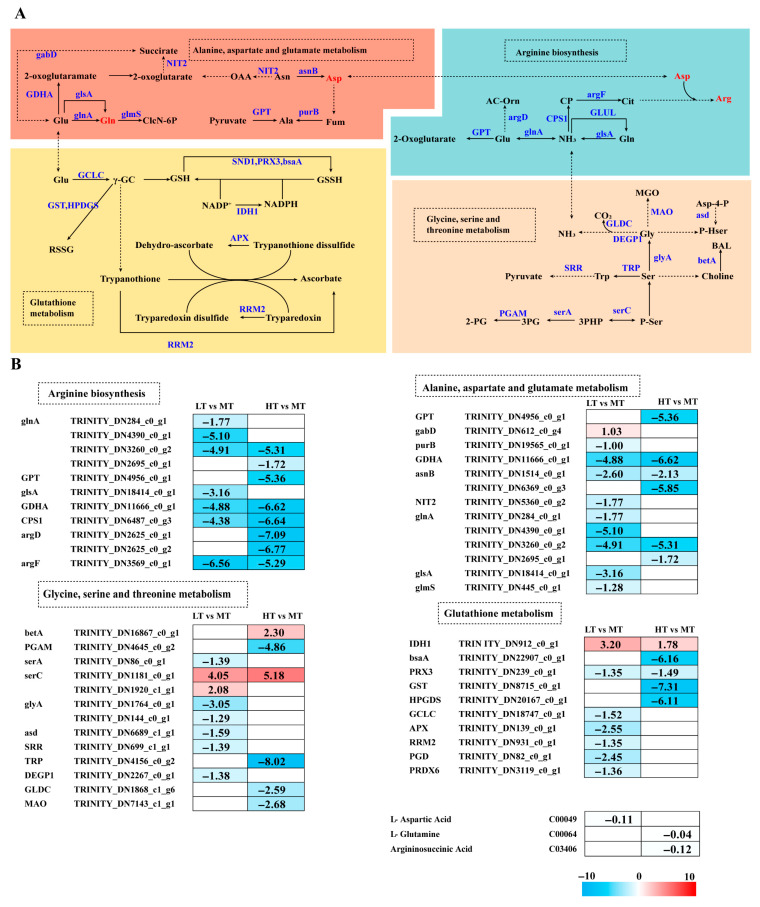
The transcriptomic and metabolomic changes in the amino acid metabolism of *B. gelatinus* under different temperature conditions. (**A**) Network diagram of amino acid metabolism pathways, different colors correspond to the distinct biological pathways, the solid arrows represent molecular interactions or relations, while the dashed arrows indicate indirect links or unknown reactions. (**B**) Differentially expressed genes (DEGs) related to amino acid metabolism. Red represents annotated differential metabolites, and blue represents annotated DEGs. The number in each cell is the log2 fold change (log_2_FC). Red and blue gradients indicate the upre-gulation and downregulation of unigenes and metabolites, respectively, while the white color represents a DEG (log_2_FC < 1 or FDR > 0.05) or a DM (FC < 1, *p*-value > 0.05 and VIP < 1). Gene abbreviations are defined in Supplementary Table S6.

Temperature stress altered amino acid metabolism in *B. gelatinus* (Figure 4A). GSH metabolism was modulated by temperature stress, with IDH1 significantly upregulated in both LT and HT conditions compared to MT. Under LT stress, most genes (*PRX3*, *GCLC*, *APX*, *RRM2*, *PGD*, and *PRDX6*) were downregulated, whereas under HT stress, except for *IDH1*, all other genes (*GPX*, *PRX3*, *GST*, and *HPGDS*) were downregulated.

Glutamine (Glu) is an essential substrate for GSH synthesis that can be converted into Gln, entering the Glu metabolic pathway. Metabolomics analysis showed Gln levels to decrease under HT stress. Under LT stress, 9 genes associated with Ala-Asp-Glu metabolism were enriched. Except for the upregulated *gabD*, all other genes (e.g., *purB*, *GDHA*, *asnB*, *NIT2*, *glnA*, *glsA*, and *glmS*) were significantly downregulated. Metabolomics data indicated downregulated Asp levels under LT stress. Compared to the MT group, all enriched DEGs (*GPT*, *GDHA*, *asnB*, and *glnA*) were downregulated under HT stress. Arginine levels were downregulated under HT stress. Genes involved in Arg biosynthesis (*glnA*, *GPT*, *CPS1*, *argD*, and *argF*) were significantly downregulated. Similarly, under LT stress, all enriched DEGs were downregulated.

The NH_3_ in Arg biosynthesis originates from *Gly* metabolism. Most genes associated with Gly-Ser-Thr metabolism (i.e., *serA*, *glyA*, asd, *SRR*, and *DEGP1*) were downregulated under LT stress. Under HT stress, *betA* and *serC* were upregulated, whereas *PGAM*, *TRP*, *GLDC*, and *MAO* showed significant downregulation. In both LT and HT conditions, *serC* was significantly upregulated (Figure 4B).

### 2.6. Effects of Temperature Stress on Porphyrin and Vitamin B6 Metabolism

**Figure 5:**
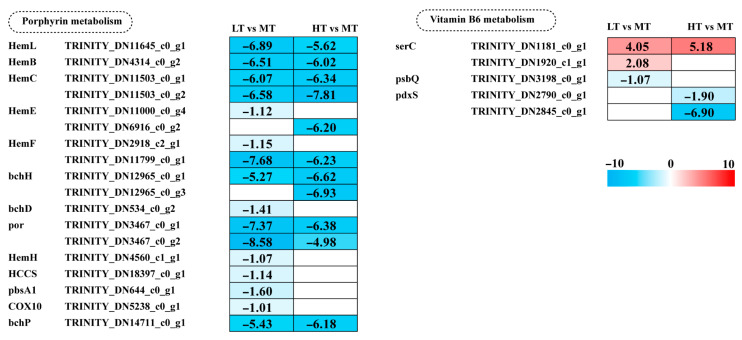
The transcriptomic and metabolomic changes in the metabolism of cofactors and vitamins of *B. gelatinus* under different temperature conditions. Red represents annotated differential metabolites, and blue represents annotated DEGs. The number in each cell is the log2 fold change (log_2_FC). Red and blue gradients indicate the upre-gulation and downregulation of unigenes and metabolites, respectively, while the white color represents a DEG (log_2_FC < 1 or FDR > 0.05) or a DM (FC < 1, *p*-value > 0.05 and VIP < 1). Gene abbreviations are defined in Supplementary Table S6.

Key genes involved in porphyrin metabolism, including most *Hem* family genes, *bchH*, por, and *bchP*, showed a significant decrease in expression in both LT and HT conditions. Additionally, five genes (*bchD*, *HemH*, *HCCS*, *pbsA1*, and *COX10*) were significantly downregulated in LT. Vitamin B6 (pyridoxal-5′-phosphate; PLP) acts as a crucial cofactor for ALAS and ALAD in porphyrin metabolism. Under LT stress, expression of the *psbQ* gene, involved in vitamin B6 metabolism, was downregulated. Under HT stress, two *pdxS* genes were downregulated. These results indicate a greater inhibition of porphyrin metabolism under LT stress, whereas high temperature stress predominantly suppresses vitamin B6 metabolism (Figure 5).

## 3. Discussion

### 3.1. Inhibition of Photosynthesis

Chl fluorescence measurements are widely used to assess photophysiological stress in marine autotrophs, with Fv/Fm and Y(II) recognized as reliable indicators of photosynthetic stress [20,21,22]. Temperature fluctuations can alter the pigment contents in algae, affecting their photosynthetic efficiency [23]. Under both LT and HT stresses, the values of Fv/Fm and Y(II) for *B. gelatinus* were inhibited to varying degrees (Figure 1A,B). Based on previous studies, we hypothesize that low-temperature and high-temperature stresses significantly inhibit the photosynthetic performance of *B. gelatinus*. Transcriptomics analysis confirmed reduced photosynthetic efficiency of *B. gelatinus*, as genes involved in photosynthetic pathways, including antenna proteins, PSII, F-type ATPase, photosynthetic electron transport, and cytochrome b6f complex, were significantly downregulated under both stress conditions (Figure 2C). Interestingly, Car content increased under both stress conditions (Figure 1D), possibly due to their function as antioxidants that mitigate photodamage and photoinhibition [24].

The light-dependent reactions of photosynthesis initiate with light absorption by antenna proteins. The results indicated that all enriched *LHCA* family genes associated with photosynthetic antenna proteins were significantly downregulated under LT/HT conditions (Figure 2C), in agreement with previous studies on temperature stress in macroalgae. For example, in *Ulva prolifera*, heat stress downregulates key photosynthesis-related genes, including several psb and *LHCA* family members, along with reduced Chl content and electron transport [25]. Similarly, after 3 h of high temperature stress, most *LHCA* genes in Sargassum have been observed to downregulate; in *K. alvarezii*, all detected genes encoding various light-harvesting complex components (e.g., *LHCA1*, *LHCA2*, and *LHCA3*) are known to downregulate under both 24 h high and low temperature stress [26,27]. In certain brown algae, specific *LHCA* genes downregulate significantly under low temperature conditions (~17 °C) [28]. Collectively, these studies suggest that downregulation of light-harvesting genes is an adaptive response to temperature stress. Additionally, in *Gracilaria bailinae*, a compensatory response has been reported in which LHCA1 is upregulated to potentially mitigate the downregulation of other antenna proteins over a 7-day high-temperature exposure period [29]. This variation may be attributed to the prolonged stress duration, likely activating protective mechanisms in the algae.

Photosystem II (PSII) is a crucial protein complex in the light-dependent reactions of photosynthesis [30]. In this study, phycobilisome (PBS) genes were significantly downregulated under both LT and HT stress (Figure 2C). Most of these genes, including *PsbS* and *PsbQ*, exhibited consistent downregulation under both LT and HT conditions, suggesting their critical roles in the responses of *B. gelatinus* to environmental stress. A previous study has reported that in *K. alvarezii* exposed to very high temperatures (~ 34 °C), genes encoding PSII subunits, including PsbA and PsbS, are downregulated [26]. Similarly, in *Agarophyton vermiculophyllum*, exposure to 36 °C for 6 h leads to downregulation of *PsbL*, *PsbM*, *PsbN*, and *PsbQ*; interestingly, *PsbX* shows significant upregulation, hinting at potential compensatory or protective mechanisms [31]. However, the present study observed no upregulated PBS genes, likely due to the short duration of temperature stress. Protective genes in *B. gelatinus* may not have been activated or reached detectable expression levels.

Electrons from PSII are accepted by the cytochrome b6f complex, where *petC* plays a crucial role in the electron transport chain [32,33]. In the HT group, genes associated with light and electron transport, including *petH*, *petF*, *petJ*, and *petC* of the cytochrome b6f complex, were significantly downregulated compared to the control, strongly inhibiting photosynthesis. Notably, *petJ* was significantly downregulated in both LT and HT groups. This consistent cross-stress regulatory pattern implies a potentially specific role of *petJ* in the stress response of *B. gelatinus*. After electron transfer through the b6f complex and establishment of a proton (H^+^) gradient, F-type ATPase synthesizes ATP, accomplishing the light-to-chemical energy conversion. Although the number of genes encoding F-type ATPases enriched in this study was relatively low (Figure 2C), this result also indicates the impact of temperature stress on algal photosynthesis. In contrast, proteomics analysis in *Pyropia yezoensis* has shown that subunits of the F-type H+-transporting ATPase are upregulated 2–6 fold under high temperature stress, supporting elevated energy demand and facilitating PSII repair during thermal adaptation [34]. This discrepancy likely reflects species-specific differences.

### 3.2. Remodelling of Carbohydrate and Energy Metabolism

Previous research has shown that changes in external temperature can alter carbohydrate and energy metabolism pathways. Gene downregulation in photosynthesis, carbon fixation, the citric acid cycle, and amino acid biosynthesis contributes to reduced ROS formation and stored energy conservation in *Picochlorum* sp. (BPE23) under 42 °C [35]. Similarly, metabolomics inhibition has been observed in several algal omics studies, including *Spirulina platensis*, *Neoporphyra haitanensis*, and *Dunaliella bardawil*. These analyses discovered that stress-induced metabolic remodelling generally conserves energy by downregulating anabolic pathways [14,36,37]. In this study, we analysed six carbohydrate and energy metabolism pathways: (i) the Calvin cycle, (ii) glycolysis/gluconeogenesis, (iii) the pentose phosphate pathway (PPP), (iv) pyruvate metabolism, (v) the TCA cycle, and (vi) fructose and mannose metabolism.

The Calvin cycle is a primary site for ROS generation [38]. In this study, all enriched DEGs in both comparison groups were found to be significantly downregulated (Figure 3B). Downregulation of key genes, such as *GAPDH* and *PGK*, results in reduced carbon assimilation capacity in *B. gelatinus*. However, these findings differ from multiple prior investigations. For instance, under elevated CO_2_ conditions, genes such as *GAPDH*, *PRI*, and *PGK* in *Phaeodactylum tricornutum* exhibit upregulation, while they downregulate when exposed to low CO_2_ conditions [39]. Studies on *Saccharina latissima* indicate that under high photosynthetically active radiation and elevated temperatures, genes related to carbon fixation are significantly downregulated. This affects both the structural components of the photosynthetic apparatus and key enzymes of the Calvin cycle [40]. Acute heat stress (~40 °C) has been observed to cause pronounced downregulation of photosynthesis-related genes, including those of the Calvin cycle [41]. Based on these research findings, Species, stress factors, and stress intensity may be causes of these differences. With changes in external environmental and nutritional conditions, cells adjust carbon flux through the Calvin cycle and other metabolic pathways, including glycolysis, the PPP, and the TCA cycle [42]. In this study, under HT stress, only the *PMM* gene involved in fructose and mannose metabolism was significantly upregulated. In contrast, other genes enriched under both low and high temperatures were significantly downregulated. In fructose and mannose metabolism, mannose is converted into fructose-6-phosphate, which then enters glycolysis. Part of its carbon skeleton is also directed into the PPP [43]. *PMM* upregulation suggests that *B. gelatinus* reprograms its cellular metabolism under HT stress, favouring glycolysis (EMP) and the PPP.

The key gene in glycolysis/gluconeogenesis, *GPI*, was significantly upregulated under stress conditions, helping the cell maintain basic metabolic flux during temperature stress. For instance, when *G. bailinae* is exposed to high temperature stress, selective upregulation of *GPI* is observed compared to the downregulation of many other carbohydrate-metabolizing enzymes. This discrepancy highlights the unique role of *GPI* under stress, potentially facilitating glycolytic intermediate conversion into substrates for other metabolic pathways [29]. However, another study has reported that *GPI* homologs exhibit temperature-sensitive expression; they are upregulated under moderate, low, and high temperatures but downregulated under freezing temperatures (5 °C) [44]. This effect may be due to differences in stress temperatures, indicating that *GPI* regulation varies with stress intensity. The pentose phosphate pathway and glycolysis operate in parallel [45]. In the LT group, five genes were upregulated in the PPP, whereas six genes were significantly downregulated. Most upregulated genes participated in the oxidative phase and helped increase NADPH production to mitigate oxidative stress. Furthermore, this effect was in good agreement with the significant increase in GSH content under LT stress (Figure 1G). Additionally, cells reduce R5P production under both LT and HT stress by downregulating non-oxidative phase genes, thereby lowering the proliferation rate.

The TCA cycle is critical to algal stress responses, enabling defence and adaptation against diverse environmental stresses [46,47]. Key genes in the TCA cycle, i.e., *LSC2* and *IDH1*, were upregulated under LT stress. At the same time, the metabolite isocitrate content decreased (Figure 3B). Under HT stress, *IDH1* expression was upregulated, suggesting that *B. gelatinus* mitigates ROS-induced damage by increasing NADPH production to sustain the GSH reduction system, paralleling the response of the PPP to temperature fluctuations. Studies on *U. prolifera* reported significant accumulation of TCA cycle-related metabolites under high temperature conditions (35 °C) [48], suggesting that factors like species and stress duration strongly influence algal temperature stress response. Additionally, D-lactate levels, involved in pyruvate metabolism, were decreased under LT stress (Figure 3B), suggesting that cells may utilize specific metabolites to meet basal energy requirements.

### 3.3. Antioxidant Effects of Amino Acid Metabolism

Temperature not only directly affects photosynthesis and respiration in algae but also significantly affects other metabolic pathways, including amino acid metabolism. Numerous studies have reported temperature-induced remodelling of amino acid metabolism, providing new molecular and metabolic insights into how algae adapt to environmental stresses [49].

GSH metabolism is a key mechanism by which large seaweeds mitigate oxidative damage. This mechanism includes detoxification by *GPX* and conjugation reactions mediated by GST, which collectively maintain cellular redox homeostasis [50]. In this study, HT stress caused significant downregulation of key genes, including GST, prx3, and GPX. Similarly, LT stress caused significant downregulation of *prx3* and *PRDX6* (Figure 4B), inhibiting the conversion of GSH to GSSH and potentially leading to GSH accumulation, consistent with results of physiological analysis (Figure 1G). In contrast to our findings, transcriptomics analysis reported significant upregulation of GST genes in *Ecklonia cava* under high temperature conditions (24 °C vs. 20 °C) [51]. Moreover, GST expression is upregulated in *S. latissima* under low temperature stress (e.g., 0 °C or 8 °C) but downregulated under moderate temperatures (~15 °C) [52]. Furthermore, GST expression is generally upregulated under heat stress in *S. japonica*, indicating that brown algae respond to heat stress by activating GSH metabolism [53]. Collectively, these studies suggest that the expression of GSH metabolism-related genes is influenced by temperature, stress duration, and species-specific responses.

GSH metabolism utilizes Glu and Gly to construct the core antioxidant system. Our results show that the expression level of the *glnA* gene, encoding Gln synthetase (involved in Glu to Gln conversion), was downregulated at HT. Metabolomics analysis further demonstrated a decrease in glutamine content under HT (Figure 4B). The expression of the NIT2 gene was significantly downregulated under LT stress, further impairing substrate production in the TCA cycle. The levels of Gln and Asp (involved in Arg biosynthesis [54,55,56]) were reduced under HT and LT conditions, respectively. The Arg biosynthesis-related DEGs, particularly *argD*, were significantly downregulated under HT. Previous studies indicate that cyanobacteria regulate Chl synthesis and nitrogen assimilation via the ArgD–Gun4 complex to maintain photosynthetic capacity under nitrogen-limited conditions [57]. Arg is also essential to the heat-tolerance mechanisms of large macroalgae [58]. The expression of the *CPS1* gene was markedly downregulated under both LT and HT conditions, suggesting its key role in Arg biosynthesis and overall abiotic stress response. Some Arg biosynthesis-related enzyme-encoding genes are upregulated in S. *japonica* under both high and low temperature stress; genomic analyses have revealed that under all stress conditions, 13 core Arg synthesis-related genes, especially the *CPS* gene, are significantly upregulated [53]. However, in this research, the expression of the *CPS* gene was notably decreased in both LT and HT conditions. Due to interspecific variation and differences in treatment duration, the defense mechanisms of *B. gelatinus* may not yet have reached the activation threshold.

The metabolic pathway serves as the primary source of NH_3_ for Arg biosynthesis and provides precursors for Glutathione (GSH) synthesis. Gly and Ser act as precursors for cell membrane phospholipids and antioxidants, and changes in their levels can directly impact membrane stability and antioxidant capacity. Thr can be converted into TH (triglycerides) and acetyl-CoA [59]. In this study, *SerC* expression was significantly upregulated, while other related genes were downregulated under both stress conditions (Figure 4B), suggesting that cells resist oxidative stress by enhancing serine synthesis for protein production and GSH biosynthesis. In contrast, *S. latissima* shows significant upregulation of Gly-Ser-Thr metabolism-related genes under low temperature (2 °C) [60], which may be attributed to differences in stress temperature. Our results indicate that *B. gelatinus* coordinates multiple amino acid metabolic pathways synergistically to resist temperature stress.

### 3.4. Inhibition of Porphyrin and Vitamin B6 Metabolism

Porphyrin metabolism is the fundamental pathway for chlorophyll and phycobilin biosynthesis. Transcriptomics analyses have provided in-depth insights into how environmental stress factors regulate gene expression in porphyrin metabolism. In *Raphidocelis subcapitata*, sulfamethoxazole exposure can significantly downregulate various genes, including *hem* family genes, which encode early steps of porphyrin biosynthesis-related enzymes, leading to a marked decline in photosynthetic capacity [61]. In the present study, the *hem* family genes were significantly downregulated under both LT and HT stress (Figure 5). Similarly, in *R. subcapitata*, erythromycin exposure can significantly downregulate *hemB*, *hemC*, and *hemE* expression, resulting in impaired photosynthetic electron transport and reduced overall cellular antioxidant capacity, indicating the critical role of porphyrin metabolism in algal stress response [62]. Furthermore, in *S. latissima*, the expression of porphyrin and chlorophyll metabolism-related genes is significantly upregulated under low temperature conditions (e.g., 2 °C and 7 °C) [60]. Notably, porphyrin metabolism-related genes are generally downregulated under antibiotic and temperature stress in these studies, suggesting that porphyrin metabolism is integral to basal cellular metabolism. Consequently, cells prioritize their suppression via a conserved regulatory mechanism to optimize resource allocation under stress.

Vitamin B6 (primarily as Pyridoxal phosphate) is an essential coenzyme in almost all living organisms, participating in multiple key biological processes, including amino acid metabolism, carbon metabolism, and signal transduction [63]. In addition, vitamin B6 possesses antioxidant properties and protects the photosynthetic apparatus from photo-oxidative stress [64]. In this study, the *pdxS* gene, which encodes a key subunit of the PLP synthesis complex, was significantly downregulated under HT conditions (Figure 5). Its downregulation can directly affect vitamin B6 metabolism and indirectly influence oxidative stress tolerance and photosynthetic efficiency, which are critical for algal survival and adaptation in variable environments [64,65]. Under low-temperature conditions, exposure to ultraviolet radiation can upregulate vitamin B6 metabolism-related genes [40]. However, in this study, PDX-related genes were not enriched under LT stress, indicating that this metabolic pathway is not regulated by a single factor. In contrast, under HT stress, PDX-related genes were significantly downregulated, indicating that algae employ multiple pathways to mitigate temperature stress-induced damage.

## 4. Materials and Methods

### 4.1. Cultivation Conditions and Experimental Design

Algae used in the present research were sourced from Haiwei Town, located in the Changjiang Li Autonomous County of Hainan Province, China. Healthy algal thalli (free of visible epiphytes and grazing marks) were selected for analysis. The algal thalli were repeatedly rinsed with sterile seawater to prevent contamination and acclimated for 7 d in sterile seawater medium. The algae were cultivated at 27 ± 0.5 °C, 30 PSU, 60 μmol photons·m^−2^·s^−1^, and a 12h light: 12h dark. After the acclimation period, 1 g fresh weight of algal tissue was cultivated in a 250 mL conical flask with sterile seawater. A year-long continuous observation of water temperature in the *B. gelatinus* cultivation farm in Haiwei Town was carried out, with measurements recorded every 15 min. During this period, the maximum and minimum temperatures recorded were 36 °C and 15 °C, respectively, although both extremes occurred only briefly. A temperature of 27 °C is found to be optimal for the growth of *B. gelatinus* [7]. The samples were subsequently exposed for 2 h to 15 °C, 27 °C, and 36 °C. Their Fv/Fm, Y(II), chlorophyll (Chl), carotenoid (Car), and GSH contents, as well as the activities of SOD, CAT, and APX, were then measured. After cultivation, the samples were collected, rapidly frozen in liquid nitrogen for 15 min, and stored at −80 °C.

### 4.2. Physical Analysis

After dark adaptation for 15 min, the maximum quantum efficiency of photosystem II (Fv/Fm) and the effective quantum yield (Y(II)) were measured using the Dual-Channel Modulated Chlorophyll Fluorometer (Dual-PAM-100, Walz, Germany) with three biological replicates [66]. The pigment contents of Chl a, Car, as well as phycobiliprotein (PE/PC) were determined using the approaches described separately by Ji et al. and Beer et al. [67,68]. Activities of the antioxidant enzymes (SOD, CAT, APX), alongside GSH and MDA levels, were assessed using kits (Solarbio, Beijing, China) following the guidelines provided by the manufacturer. All measurements were performed with three biological replicates.

### 4.3. Transcriptome Analysis and Quantitative Real-Time Polymerase Chain Reaction (qRT-PCR) Analysis

Approximately 0.5 g of fresh samples was weighed and thoroughly ground in liquid nitrogen. Subsequently, QIAzol Lysis Reagent kit (Qiagen, Hilden, Germany) was utilized for the isolation of total RNA. Library preparation and sequencing services were provided by Shanghai Majorbio Bio-pharm Biotechnology Co., Ltd. (Shanghai, China). De novo assembly of clean reads derived from all algal samples was implemented using Trinity [69]. Following optimization of the assemblies using TransRate and CD-HIT (v4.5.7) [70,71], quality evaluation was conducted via BUSCO (v3.0.2) [72]. The optimized assemblies were annotated by comparison using six major databases (DBs), including the NR, Swiss-Prot, Pfam, EggNOG, GO, and KEGG, and annotation statistics were generated for each. Transcript abundance was quantified using RSEM software (v1.3.1) [73], with reads aligned to the assembled sequences and unigene abundance expressed as FPKM values. Sample reproducibility and quality were evaluated using principal component analysis (PCA) and Pearson correlation analysis. Differentially expressed genes (DEGs) were identified using DESeq2 (1.42.0) [74], with significance thresholds of FDR < 0.05 and |log_2_FC| ≥ 1. GO and KEGG pathway enrichment analyses of DEGs were performed using GOATOOLS (v1.4.4) [75] and the Python SciPy package (https://scipy.org/install/ (accessed on 24 December 2025)), respectively, with an adjusted *p*-value of < 0.05.

To confirm the transcriptomic findings, nine DEGs from major metabolic pathways such as photosynthesis, carbohydrate metabolism, nitrogen metabolism, amino acid biosynthesis, and signal transduction were randomly chosen for qRT-PCR validation. A plant total RNA extraction kit was employed for the extraction of total RNA. Following concentration normalization, 12 μL of RNA (500 ng–2 μg) was used for reverse transcription. Reverse transcription was implemented with the HiScript^®^ Q RT SuperMix for qPCR kit (Vazyme, Nanjing, China) in a 20 μL reaction system. Supplementary Table S7 provides the primers utilized for the qRT-PCR experiment. The qPCR primers were designed based on the gene sequence information using Primer 5 software (v5.0). The cDNA products were quantified using the Pharmaceutical Analytics QuantStudio™ 5 Real-Time PCR System (Applied Biosystems, Foster City, CA, USA). Gene expression levels were determined using the 2*^−^*^ΔΔ*CT*^ method [76], where the internal reference was the 18S rRNA gene of *B. gelatinus*.

### 4.4. Metabolomic Analysis

For metabolomics analysis, 50 mg of freeze-dried sample was transferred to a 2 mL tube containing grinding beads and mixed with 400 μL of extraction solvent. The mixture was ground in a frozen tissue grinder at 50 Hz for 6 min and ultrasonically extracted at 5 °C and 40 Hz for 30 min. The LC-MS/MS analysis of sample was conducted on a Thermo UHPLC-Q Exactive HF-X system at Majorbio Bio-Pharm Technology Co., Ltd. (Shanghai, China). for LC-MS/MS analysis. The Progenesis QI software (Waters Corporation, Milford, CT, USA) was utilized to process the raw LC-MS data by performing baseline filtering, detecting peaks, integrating data, correcting retention times, and aligning peaks. The MS and MS/MS spectra were matched against public DBs, including HMDB, Metlin, and a proprietary database from custom-built database (Majorbio, Shanghai, China), for metabolite identification. The pre-processed data matrix was then analysed using the ropls package v1.6.2 in R to perform PCA and orthogonal partial least squares-discriminant analysis (OPLS-DA). Seven-fold cross-validation was conducted to assess model stability. Significantly differential metabolites were screened for by applying thresholds of variable importance in projection (VIP) > 1 from the OPLS-DA model and a *p*-value < 0.05 from Student’s *t*-test. Differential metabolites were annotated using the KEGG database to identify their associated pathways. Pathway enrichment analysis was performed using the Python package scipy.stats(v1.0.0), and Fisher’s exact test was performed to identify relevant biological pathways.

### 4.5. Statistical Analysis

All results were reported as means with their corresponding standard deviations. Statistical analyses were conducted in IBM SPSS Statistics 25.0, applying one-way ANOVA to assess treatment effects. Post hoc comparisons were performed using Duncan’s test at a significance threshold of *p* < 0.05.

## 5. Conclusions

In this research, we examined the physiology, transcriptome, and metabolome of *B. gelatinus* when subjected to temperature stress, with a focus on stress-related genes and metabolic pathways. Our results revealed that photosynthesis, antioxidant systems, amino acid metabolism, carbohydrate metabolism, cofactor and vitamin metabolism were involved in the temperature stress response in *B. gelatinus*. Under temperature stress, photosynthesis, photobiological carbon fixation, Arg biosynthesis, porphyrin metabolism, and vitamin B6 metabolism were completely inhibited, while other carbohydrate and amino acid metabolism pathways underwent varying degrees of restructuring. In addition, *Psb*, *GPI*, *GAPDH*, *PGK*, *GST*, and *GPX* genes were found to play a key role in temperature stress resistance in *B. gelatinus*.

## Data Availability

The original contributions presented in this study are included in the article/Supplementary Materials. Further inquiries can be directed to the corresponding author.

## Supplementary Materials

The following supporting information can be downloaded at: https://www.mdpi.com/article/10.3390/ijms27020593/s1.
