# Supplementary material for: Physiological and Molecular Response Mechanisms of Betaphycus gelatinus to Low- and High-Temperature Stress
Source: Int J Mol Sci. 2026 Jan 7;27(2):593. doi: 10.3390/ijms27020593 (PMC12841325; doi:10.3390/ijms27020593)
Supplement: Supplementary file 1 [file ijms-27-00593-s001.zip › Supplementary Figure.pdf]

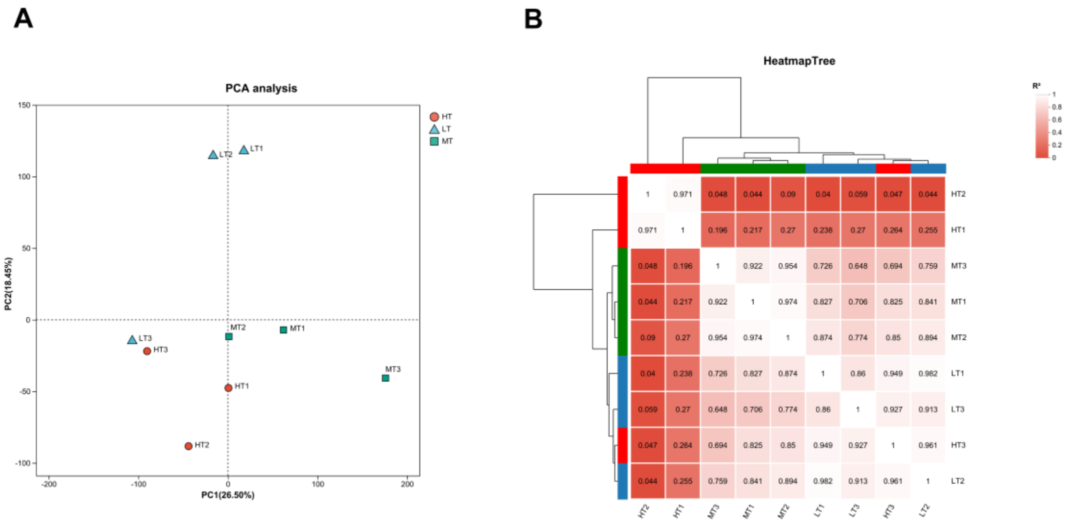

Supplementary Figure S1. Transcriptome analysis in *B. gelatinus* under different temperature conditions. (a) Principal component analysis of transcriptome samples, (b) Correlation heatmap of transcriptome samples.

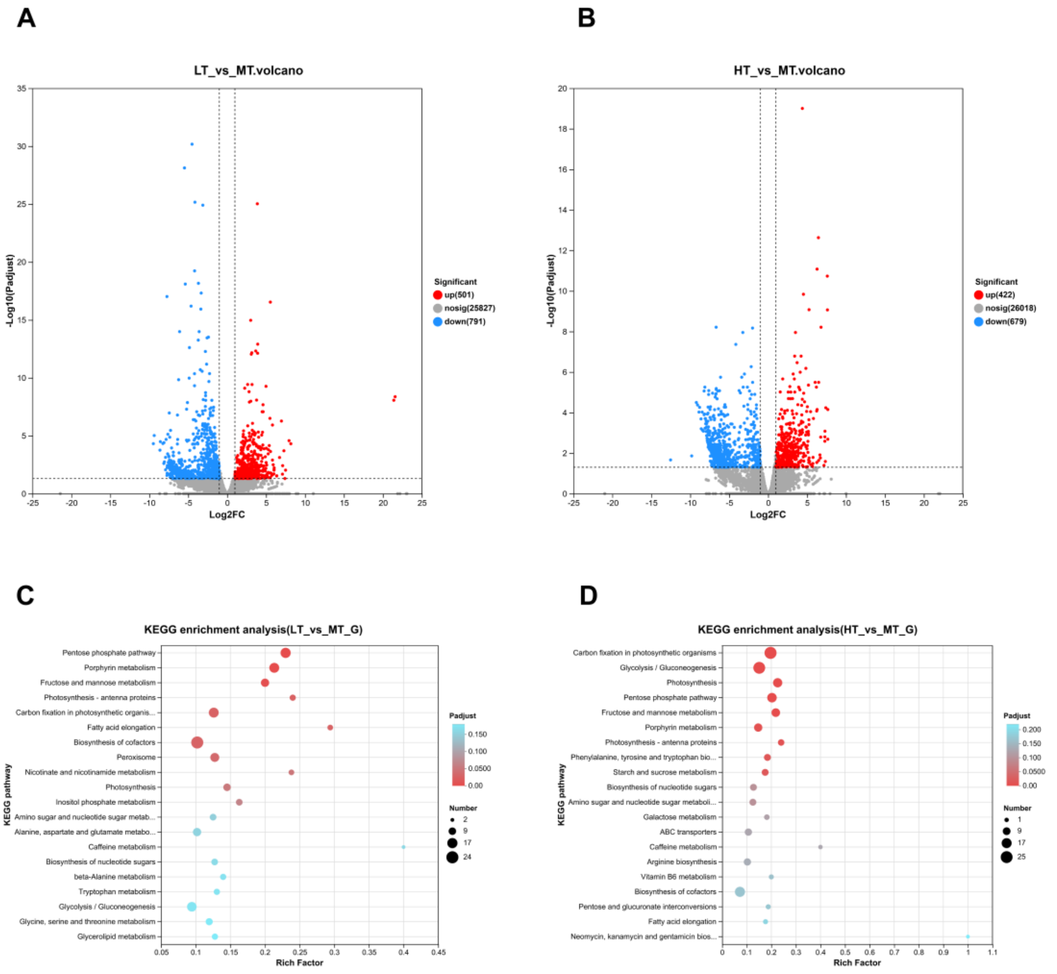

Supplementary Figure S2. Volcanic maps and bubble plot of KEGG enrichment for DEGs. (A and C) volcanic maps and bubble plot of KEGG enrichment for DEGs in LT vs MT, (B and D) volcanic maps and bubble plot of KEGG enrichment for DEGs in HT vs MT.

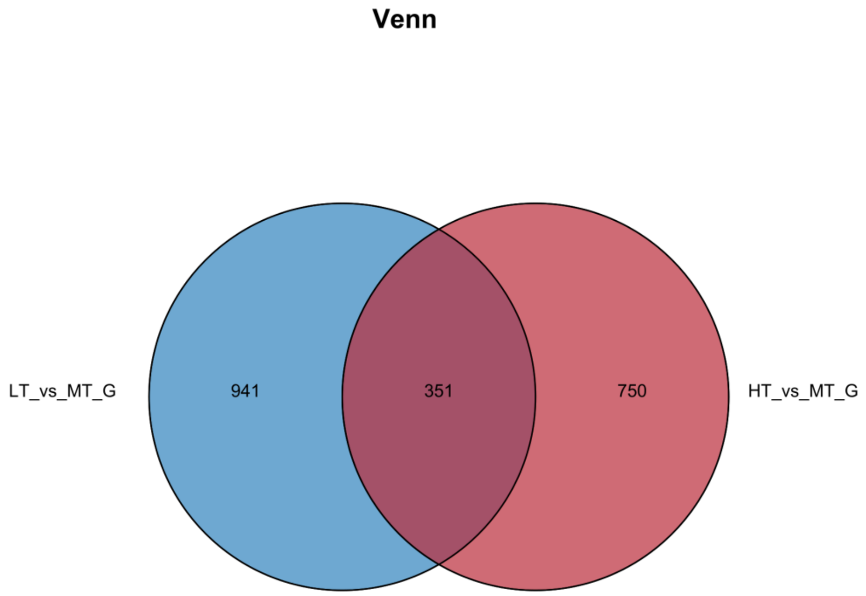

Supplementary Figure S3. Venn diagram of s (DEGs) under different temperature conditions of *B.gelatinus*.

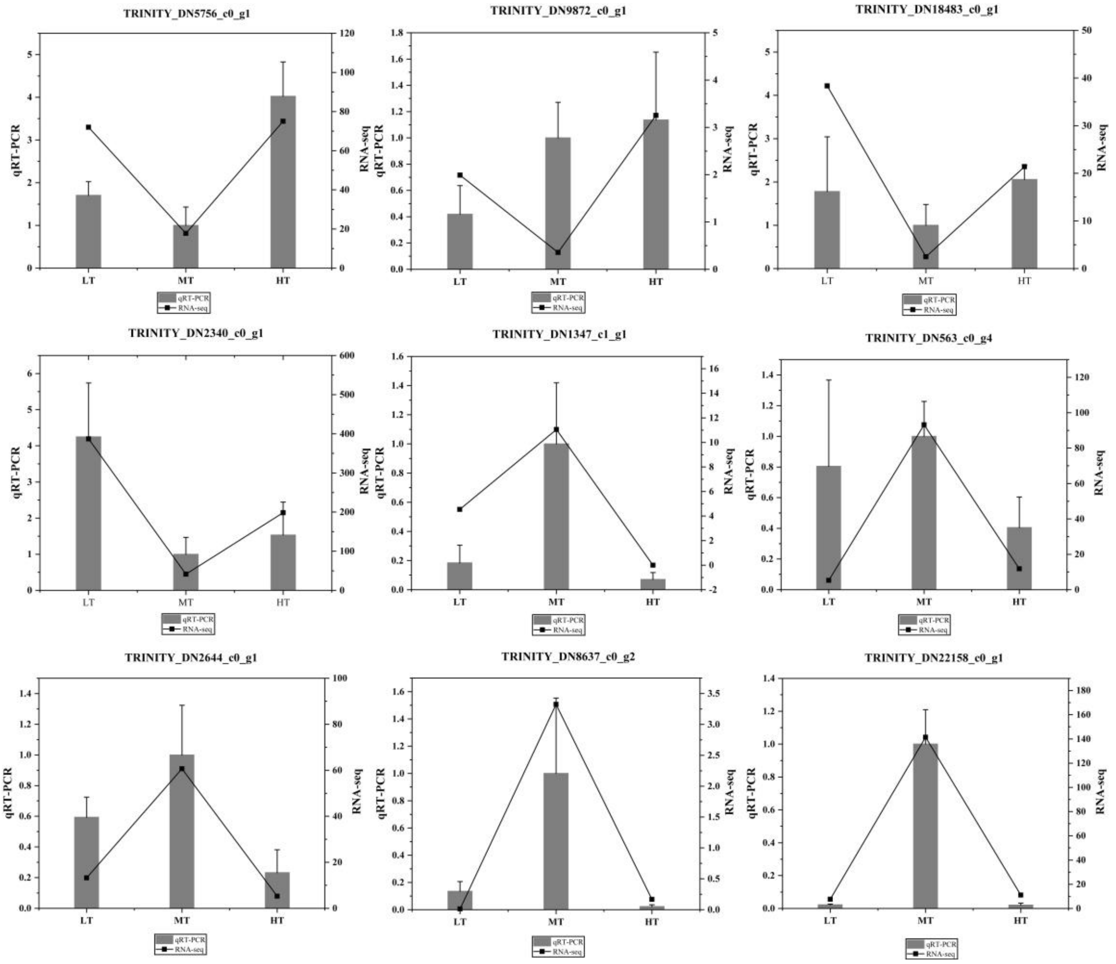

Supplementary Figure S4. Real-time quantitative PCR results. The bar graphs represent the qRT-PCR data, the line plots represent the RNA-Seq data. Nine genes were randomly selected, including TRINITY_DN5756_c0_g1, TRINITY_DN9872_c0_g1, TRINITY_DN18483_c0_g1, TRINITY_DN2340_c0_g1, TRINITY_DN1347_c1_g1, TRINITY_DN563_c0_g4, TRINITY_DN2644_c0_g1, TRINITY_DN8637_c0_g2 and TRINITY_DN22158_c0_g1.

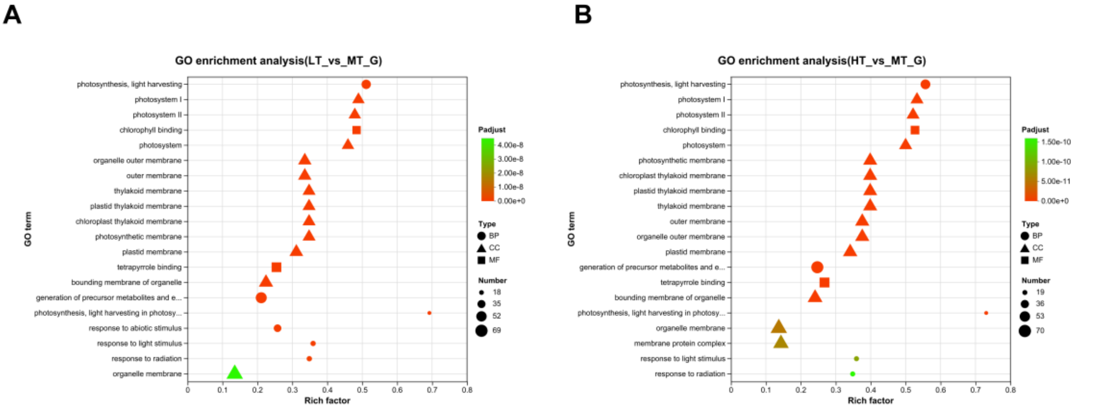

Supplementary Figure S5. GO enrichment bubble chart of differentially expressed genes between (A) (LT vs MT) group and (B) (HT vs MT) group in transcriptome analysis.

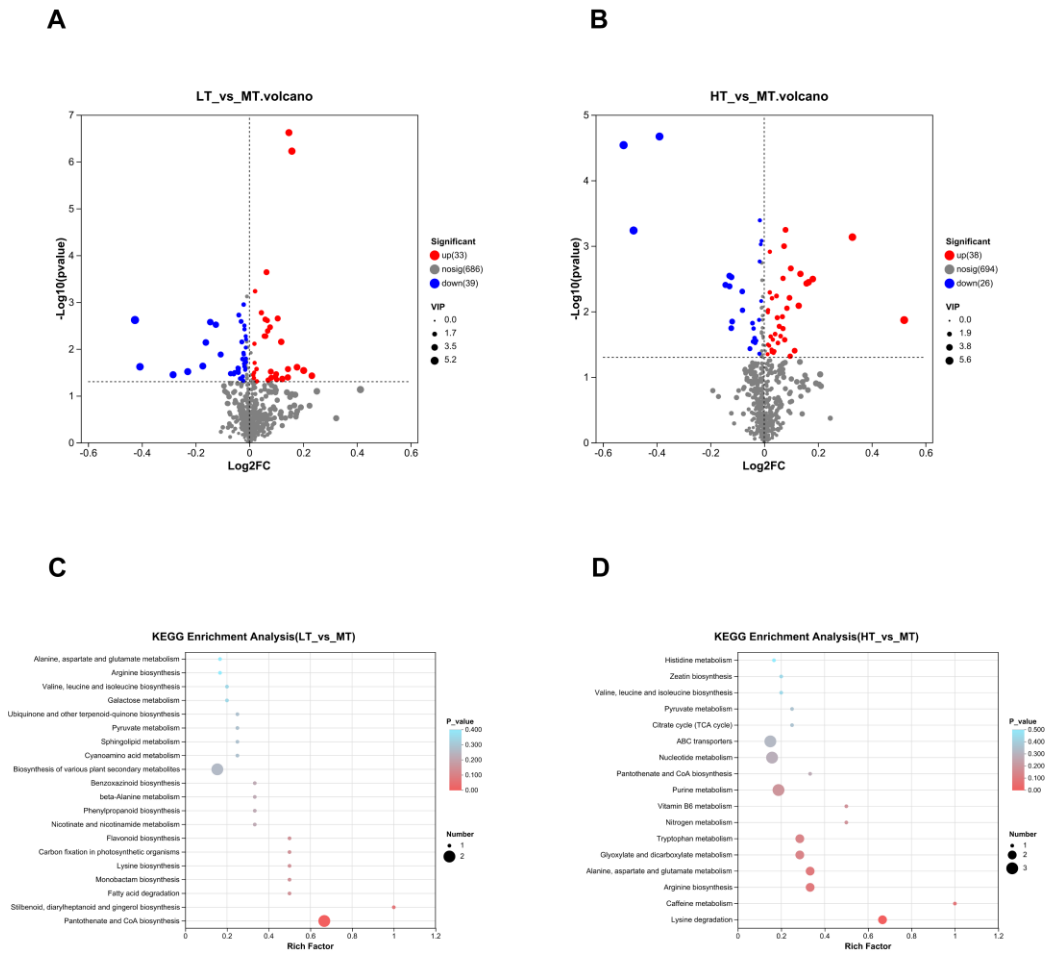

Supplementary Figure S6. Volcanic maps and bubble plot of KEGG enrichment for DAMs. (A and C) volcanic maps and bubble plot of KEGG enrichment for Dams in LT vs MT, (B and D) volcanic maps and bubble plot of KEGG enrichment for DAMs in HT vs MT.

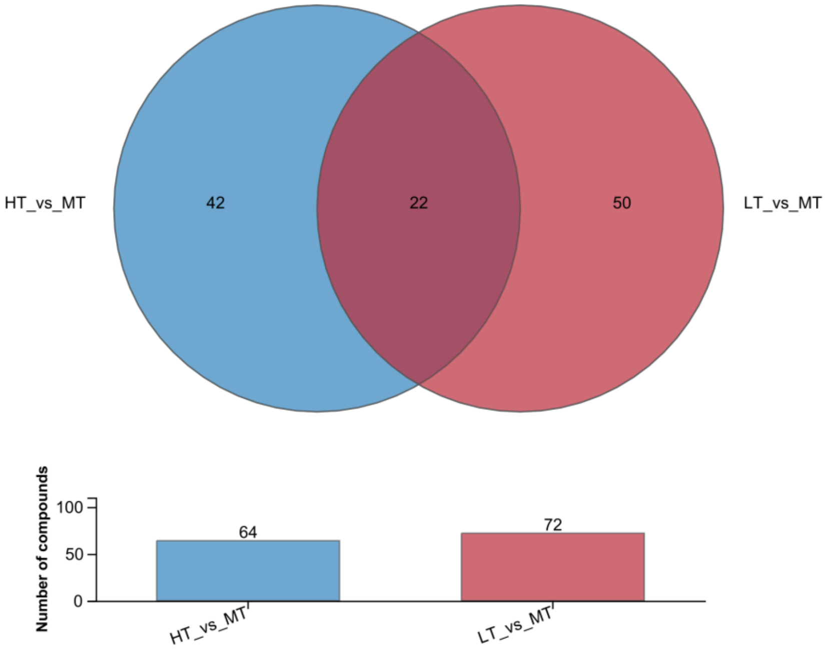

Supplementary Figure S7. Venn diagram of s (DAMs) under different temperature conditions of *B.gelatinus*.
